# Antenatal care satisfaction in a developing country: a cross-sectional study from Nigeria

**DOI:** 10.1186/s12889-018-5285-0

**Published:** 2018-03-20

**Authors:** Dumbiri J. Onyeajam, Sudha Xirasagar, Mahmud M. Khan, James W. Hardin, Oluwole Odutolu

**Affiliations:** 1 Department of Health Services Policy and Management, Arnold School of Public Health, University of South Carolina, 915 Greene Street, Ste 360, Columbia, SC 29208 USA; 2 Biostatistics Division, Department of Epidemiology and Biostatistics, Arnold School of Public Health, University of South Carolina, Columbia, SC 29208 USA; 3 Nigeria Country Office, The World Bank, Abuja, Nigeria

**Keywords:** Patient satisfaction, Antenatal care, Free care, Provider behavior, Provider communication skill, Availability of equipment, Ease of access to medications, Developing country

## Abstract

**Background:**

Utilization of Antenatal Care (ANC) is very low in Nigeria. Self-reported patient satisfaction may be useful to identify provider- and facility-specific factors that can be improved to increase ANC satisfaction and utilization.

**Methods:**

Exit interview data collected from ANC users and facility assessment survey data from 534 systematically selected facilities in four northern Nigerian states were used. Associations between patient satisfaction (satisfied, not-satisfied) and patient ratings of the provider’s interactions, care processes, out-of-pocket costs, and quality of facility infrastructure were studied.

**Results:**

Of 1336 mothers, 90% were satisfied with ANC. Patient satisfaction was positively associated with responsive service (prompt, unrushed service, convenient clinic hours and privacy during consultation, AOR 2.42, 95% CI 2.05–2.87), treatment-facilitation (medical care-related provider communication and ease of receiving medicines, AOR 2.03, 95% CI 1.46–2.80), equipment availability (AOR 1.10, 95% CI 1.01–1.21), staff empathy (AOR 1.82, 95% CI 1.03–3.23), non-discriminatory treatment regardless of patient’s socioeconomic status (AOR: 1.87, 95% CI 1.09–3.22), provider assurance (courtesy and patient’s confidence in provider’s competence, AOR 1.48, 95% CI 1.26–1.75), and number of clinical examinations received (AOR 1.28, 95% CI 1.10–1.50). ANC satisfaction was negatively impacted by out-of-pocket payment for care (vs. free care, AOR 0.44, 95% CI 0.23–0.82).

**Conclusions:**

ANC satisfaction in Nigeria may be enhanced by improving responsiveness to clients, clinical care quality, ensuring equipment availability, optimizing easy access to medicines, and expanding free ANC services.

**Electronic supplementary material:**

The online version of this article (10.1186/s12889-018-5285-0) contains supplementary material, which is available to authorized users.

## Background

Antenatal care (ANC) utilization rate in Nigeria (a lower-middle income country) is quite low, about 61% of pregnant women visited a skilled provider at least once during their pregnancy compared with the documented average of 79% for all lower-middle income countries [1, 2]. ANC enables effective management of pre-natal morbidities, and may facilitate institutional delivery and postpartum care, thereby improving maternal and new-born health outcomes [3–5]. In Nigeria, 41% of women who utilized skilled ANC did not deliver in a healthcare facility [1, 3]. Studies suggest that dissatisfaction with the ANC experience may partly explain this low level of institutional delivery by ANC users [6, 7]. Consistent with low antenatal care and institutional delivery rates in Nigeria (36%), maternal outcomes are poor [1, 2, 8]. Nigeria ranks among the top 16 nations in maternal mortality, 576 deaths per 100,000 live-births [1, 2, 8]. With just 2.45% of the world’s population, Nigeria accounts for 19% of maternal deaths [2, 8]. Many developing countries have successfully reduced maternal mortality by expanding maternal service utilization through policy innovations [9, 10].

Policies to maximize patient satisfaction at ANC visits may translate into sustained ANC use throughout the pregnancy and increased rates of institutional delivery. The role of health facility and staff characteristics in general outpatients’ satisfaction with care is well documented and includes facility infrastructure and amenities availability (equipment, drugs, comfortable waiting area), interpersonal interactions of staff and providers (e.g. courtesy, empathy), provider technical performance, care logistics and the absence of financial barriers to care [11–13]. The context of patient satisfaction with ANC may be different compared to sick outpatient care because of population perception of (1) low utility of ANC and (2) the opportunity cost of time and effort spent on a preventive service such as ANC in the context of others survival priorities of the poor in many developing countries [14].

Government facilities are the principal source of care for the Nigerian population, particularly in rural areas. There is no study of the role of out-of-pocket expenditures and patient-experienced access to medications in ANC satisfaction at government health facilities [15–19]. From patients’ perspective, the role of out-of-pocket expense in patient satisfaction is important as living expenditure competes with preventive maternal healthcare need for household income [14]. Further, despite officially free services in some states, patients’ access to medicines, may be limited due to non-availability of medicines, apathetic pharmacy and facility staff, and procedural complexities in receiving the drugs [12, 20]. This study identifies some policy-modifiable structural factors (availability of equipment, qualified providers, and out-of-pocket cost) and process of care factors (clinical examination, staff responsiveness, care communication) driving ANC patients’ satisfaction with care (a measures of their judgement on quality of overall care experience) at government facilities. These factors may play a role in ANC utilization of specific demographic segments of the population [3, 21].

## Methods

We conducted a cross-sectional study, using the World Bank-assisted, Nigeria State Health Investment Project (NSHIP) baseline survey data. The NSHIP surveyed functional government health facilities in six states (3 project states selected for strengthening maternal and child health services infrastructure based on administrative considerations and 3 control states [one state matching each project state on regional location and demographic characteristics], out of 36 states and the federal capital). The survey included exit interviews of ANC outpatients in 2013–2014. We used baseline survey data when the NSHIP project interventions were not yet implemented. Our study used data from four northern states, namely, Adamawa, Nasarawa, Benue and Taraba. These states had a documented skilled ANC utilization rate of 68%, (with 73% of utilizers having ≥ 4 visits) in a separate community survey, similar to the national average (61% skilled ANC use, and 81% of them having ≥ 4 visits).

The NSHIP survey used multistage sampling to select health facilities using state administrative divisions (Local Government Areas [LGAs] and geographic wards) as the strata. In Adamawa and Nasarawa (intended intervention states), all LGAs and wards were selected and in the remaining two states, a random sample of LGAs, and then all wards in the selected LGAs were sampled. Within each ward, all state-owned hospitals (as available), and a random sample of functioning government-owned primary care facilities (those with consistent maternal and child health service utilization in the prior year) were surveyed. Surveyors collected facility-level data on the availability and functionality of infrastructure and manpower. A total of 84 surveyors (40-Adamawa, 20-Nasarawa, 16-Tarabae, 8-Benue, allocated according to workload) were trained in data collection and interviewing skills for the project. Facility-level variable values (objectively scored by surveyors after direct observation) are common to patients interviewed at the facility. At each facility, three ANC outpatients were interviewed (the first three to exit the facility during surveyors’ visit). Interviews were conducted at preselected designated exit areas within the facility that ensured privacy and confidentiality. If a patient declined, the next eligible exiting patient was interviewed up until 3 patients were interviewed, the close of ANC clinic time or end of survey time designated for a facility. Note, not all facility had ANC patients visiting on day of survey. Surveyors documented written informed consent. Survey questions covered patient socio-demographics, and their perceptions of care access, provider-patient interactions, out-of-pocket expenditure for care and satisfaction with services. A Hausa-translated survey format was also offered (the major language in northern Nigeria). The study was approved by the University of South Carolina Institutional Review Board.

Our unit of analysis is the ANC outpatient. All interviewed ANC patients were eligible for inclusion in the study (2–3/facility). The primary outcome of interest is the patient’s satisfaction with ANC (satisfied, not satisfied). We adjusted for sociodemographic variables influencing use of maternal healthcare - age (years), education (less than or some secondary education, high school diploma or higher), marital status (married/living-together, other), and household wealth quartile (poor, lower-middle, upper-middle, rich, computed by principal component analysis of patient-reported household assets) [3]. We also adjusted for parity (primi, multigravida), and prior ANC visit to the facility (yes/no) [3].

Facility-level independent variables of interest are surveyor-assessed infrastructure (cleanliness and amenities, general medical care equipment, and essential drugs), and staff availability (percent of employed clinical staff present on the survey date). General medical care equipment included essential ANC items, such as adult weighing scale, height measure, thermometer, blood pressure meter, stethoscope, otoscope, fetoscope, etc. Each item was scored 1 if available and functional, and added to produce the facility’s equipment score. Similarly, the facility cleanliness and amenities score was the sum of scores on clean waiting area, protection from weather elements, fan/AC, adequate seating (no patients standing), clean restrooms, clean environment, consulting room privacy, untorn beds, and adequate lighting. The drug availability score was the sum of essential drugs available on the day of survey (at least one dose, no stock-out in the prior 30 days). Surveyed drugs included antibiotics, vitamins and minerals, antihistamines, analgesics, antimalarials, antihypertensives, diagnostic kits, and emergency obstetric drugs.

The patient survey items used to capture patient-level independent variables below are presented in Additional file 1: Table S1 “Pregnant Women’s Satisfaction with their Antenatal Care Visit - Survey Instrument”. Based on the total amount spent on registration, laboratory, ultrasound, medicines, and any informal fees, financial access (out-of-pocket expenditure) was measured as free vs. paid care towards ANC visit. Geographic access measured travel distance from home (< 3 km, ≥ 3 km). Exploratory factor analysis (principal factor method, promax rotation, and simple structure) yielded two factors from 10 items on provider-patient interactions (Likert scale, agree, neutral, disagree). The two factors are: assurance of providers and responsiveness of the facility to patients. The rotated factor pattern and loadings are presented in Table 1. The factor items are consistent with internationally documented, affective elements of outpatient experience [11]. Item scores were added to compute factor scores. In addition, four composite variables were constructed based on intuitive assessment of items cohesiveness: treatment-facilitation, clinical examinations, Maternal and Child Health (MCH) counselling, and preventive medication. Treatment-facilitation was the sum of item scores on the provider’s effectiveness of medical care-related communications and patient-perceived ease of access to medications. These items were combined because collectively they represent key patient goals in a medical encounter and may impact patients’ understanding of and adherence to treatment, medical outcome, and patient satisfaction [22, 23]. The clinical examination score was the sum of patient-reported examinations received (weight, height, blood pressure, uterine height, urine test, blood test and abdominal examination). The MCH counselling score was the sum of informational items received: dietary advice, danger signs during pregnancy, family planning, breast feeding, HIV, and delivery care plan. The preventive medication score was the sum of preventive medications received: iron/folic acid supplement, antimalarial pills, and tetanus toxoid (pill possession was verified). Finally, stand-alone items that did not load on a factor were used: empathic provider (patient-perceived caring attitude of staff, yes/no), and non-discriminatory care behavior (perception of care provided without socioeconomic status-based discrimination, yes/no).

**Table 1:** Factor loadings of items measuring perceived quality of patient-provider interactions (exploratory factor analysis, promax rotation)

Items	Standardized Coefficient	
	Factor 1	Factor 2
The health staff are courteous and respectful	**0.31**	0.15
The health workers in this facility are extremely thorough and careful.	**0.42**	0.16
You trust in the skills and abilities of the health workers of this facility.	**0.63**	0.00
You completely trust the health worker’s decisions about medical treatments in this facility.	**0.59**	−0.04
The health workers in this facility are very friendly and approachable.	**0.61**	− 0.09
The health workers in this facility are easy to make contact with.	**0.35**	0.19
The amount of time you spent waiting to be seen by a health provider was reasonable.	−0.02	**0.40**
You had enough privacy during your visit.	−0.04	**0.46**
The health worker spent a sufficient amount of time with you	0.01	**0.60**
The hours the facility is open are adequate to meet your needs	−0.02	**0.58**

Minimum factor loading coefficient set at 0.30

Factor 1: Assurance (Cronbach alpha, reliability coefficient: 0.70)

Factor 2: Responsiveness (Cronbach alpha, reliability coefficient: 0.57)

Inter-factor correlation: 0.55

For 77 facilities with missing drug availability data, we imputed data by multivariate normal regression analysis using the facility scores on cleanliness and amenities, general medical equipment, and availability of employed clinical staff as predictor variables. Multiple imputations predict missing data values based on available data to produce stable estimates [24]. We used 50 imputations (exceeding the percentage of facilities with missing data, 14.4%), and assumed that data were missing at random (MAR). Because the observed and imputed data distributions did not differ based on visual comparison of the plots, the imputation model was considered acceptable for these variables. This was a pragmatic decision rule used to determine the reliability of the imputations. The same approach was applied to impute missing data on staff availability. Imputed values were deemed unreliable and rejected because the observed and imputed data distributions were different. Therefore, facilities with missing staff availability were excluded from data analysis.

We conducted univariate analysis to describe the patient and facility samples, and bivariate analysis (chi-square/t-tests) to study unadjusted associations of the independent variables with patient satisfaction. Testing for multicollinearity using variance inflation factors indicated that correlations among the explanatory variables were not a concern. We used multilevel logistic regression modeling, accounting for clustering of patients within facilities, to study factors associated with ANC satisfaction. All independent variables capturing the structure and process of care discussed above and consistent with the Donabedian framework of healthcare quality which could be measured as patients’ judgment on care received (patient satisfaction) were included in the initial model, adjusting for potential maternal socio-demographics as covariates [21]. We used manual, backward selection process to progressively exclude non-significant variables (*p* > 0.10), and tested for goodness of fit with Wald test statistics. We verified the final model fit using the Hosmer-Lemeshow goodness-of-fit test (9.40, *p* value of 0.31). A *p*-value of 0.05 was used for statistical significance. Stata version 14 was used for analysis.

## Results

Of 826 selected health facilities, 717 were surveyed, out of which 554 facilities had ANC patient survey data available on 1438 patients. Of 1438 interviewed ANC patients, we excluded 102 patients with missing data (6 on age, 51 on the response about non-discriminatory treatment, and interviewees from 45 facilities with missing data on staff availability (imputation was deemed unreliable). The final analytical sample consisted of 1336 ANC patients (93% of interviewee) attending 534 health facilities (range 2–3 ANC patients/facility).

Table 2 presents the distributions of the health facility variables, showing generally poor infrastructure - general medical care equipment (on average, 6.40 items available out of 23), drugs (13 out of 48), and general cleanliness and amenities (6.35 out of maximum possible score of 11). Table 3 presents the sample distribution of the1336 ANC outpatients with a mean age of 25 years, and the majority married or living together (96%), generally less educated (85% with primary or no formal education), had a previous pregnancy experience (63%), paid for care (71%), and satisfied with the ANC visit (90%). The number of essential ANC services received by surveyed patients, on average, was inadequate: clinical examinations (4.64 examinations out of 7 expected), MCH counselling (3.16 out of 6), and preventive medications (1.93 out of 3). A significant proportion of patients reported unfavorable staff attitudes; discriminatory behaviors, 36%, and non-empathic providers, 30%. Regarding other perceptions, the scores were generally high (responsive service, 7.20 out of a maximum possible score of 8; provider assurance, 11.40 out of 14; treatment-facilitation, 3.70 out of 4).

**Table 2:** Healthcare facility variables summary scores, and bivariate associations with ANC patient satisfaction at the facility, northern Nigeria. *N* = 534

Facility structural characteristics^a^	Mean (std.dev)	Maximum expected score	Number
Availability of general-care equipment^b^	6.38 (4.34)	23	534
Availability of drugs^b^	12.98 (9.57)	48	457
Proportion of employed clinical staff available on day of survey (%)^c^	75.40 (25.1)	100	534
Facility cleanliness and amenities	6.35 (2.77)	11	534

*ANC* Antenatal Care

^a^Indicators are objectively measured by surveyors

^b^Significantly associated with satisfaction in bivariate analysis at *p* < 0.05

^c^Clinical staff: Doctors, nurses, midwives, auxiliary nurse, pharmacists, laboratory technologists, technicians, community health officers, community health extension workers

**Table 3:** ANC patients’ sociodemographic distribution and reported care experience by satisfaction with care, Northern Nigeria. *N* = 1,336^a^

	Total n(%)	Satisfied n(%)	Not satisfied n(%)	*p*-value
Total Respondents	1336 (100)	1204 (90)	132 (10)	
Sociodemographic and Maternal Factors				
Age (years)	24.7 (6)	24.6 (6)	25.6 (6)	0.97
Marital status				
Married/living-together	1286 (96)	1161 (90)	125 (10)	0.32
Others	50 (4)	43 (86)	7 (14)	
Education				
Less than secondary	1135 (85)	1024 (90)	111 (10)	0.77
Secondary or higher	201 (15)	180 (90)	21 (10)	
Wealth quartile				
Poor	333 (25)	300 (90)	33 (10)	0.24
Lower-middle	335 (25)	306 (91)	29 (9)	
Upper-middle	333 (25)	291 (87)	42 (13)	
Rich	335 (25)	307 (92)	28 (8)	
Gravida status^b^				
Primigravida	494 (37)	447 (91)	47 (9)	0.73
Multigravida	842 (63)	757 (90)	85 (10)	
Previous ANC in visited facility				
Yes	975 (73)	884 (91)	91 (9)	0.27
No	361 (27)	320 (89)	41 (11)	
Healthcare Access Factors				
Distance travelled (km)				
< 3	1223 (92)	1099 (90)	124 (10)	0.30
≥ 3	113 (9)	105 (93)	8 (7)	
Out-of-pocket expenditure (Naira)				
Free (no payment)	392 (29)	354 (90)	38 (10)	0.88
100–1000	832 (62)	747 (90)	85 (10)	
> 1000	112 (8)	103 (92)	9 (8)	
Patient Care Experience				
Assurance (mean, SD)^c^	11.4 (1.20)	11.5 (1.06)	10.0 (1.80)	0.00
Responsiveness (mean, SD)^d^	7.2 (1.30)	7.5 (1.00)	5.2 (1.60)	0.00
Treatment-facilitation (mean, SD)^e^	3.7 (0.70)	3.8 (0.60)	3.12 (1.10)	0.00
Clinical examinations received (mean, SD)^f^	4.64 (1.71)	4.72 (1.70)	3.94 (1.59)	0.00
Maternal and child health counselling items (mean, SD)^g^	3.16 (1.90)	3.25 (1.89)	2.37 (1.73)	0.00
Preventive medications received (mean, SD)^h^	1.93 (0.91)	1.92 (0.91)	2.05 (0.92)	0.95
Non-discriminatory treatment regardless of socioeconomic status				
Yes	853 (64)	792 (93)	61 (7)	0.00
No	483 (36)	412 (85)	71 (15)	
Empathic providers				
Yes	933 (70)	883 (95)	50 (5)	0.00
No	403 (30)	321 (80)	82 (20)	

*ANC* Antenatal Care

^a^Differences in the distributions of satisfactory and non-satisfactory ANC are significant at *p* < 0.05

^b^Gravida status: Primigravida-first pregnancy, Multigravida-second or higher

^c^Assurance: Provider courtesy and accessibility, and trust in provider’s skill and treatment decisions

^d^Responsiveness: Wait time, unrushed consultation, privacy during care, and clinic service hours

^e^Treatment-facilitation: Effective provider communication regarding maternal and neonatal health condition and treatment, and ease of access to prescribed drugs

^f^Clinical examination score: Measurement of weight, height, blood pressure, uterine height, urine test, blood test, and abdominal examination

^g^Maternal and child health counselling score: Counselling on diet, danger signs during pregnancy, family planning, breast feeding, HIV and delivery care plan

^h^Preventive medications score: Receipt of iron/folic acid supplement, antimalarial, and tetanus toxoid

Table 4 presents the factors associated with patient satisfaction, adjusted for demographic variables. Paid care was associated with lower odds of satisfaction (AOR 0.44 relative to free care, 95% CI 0.23–0.82). Per patients’ perspective on quality, each unit increase in provider assurance score was associated with significantly higher odds of satisfaction (AOR: 1.48, 95% CI: 1.26–1.75), as also responsive service (AOR: 2.42, 95% CI: 2.05–2.87), and so was treatment-facilitating climate (AOR 2.03, 95% CI: 1.46–2.80). Provider concern for patients’ wellbeing was associated with higher odds of satisfaction (patients’ perception of empathic provider - AOR: 1.82, 95% CI: 1.03–3.23), as also a perception of being treated without discrimination based on socioeconomic status (AOR: 1.87, 95% CI: 1.09–3.22). Each additional clinical examination received (reported by patients) was associated with 28% higher odds of satisfaction (AOR: 1.28, 95% CI: 1.10–1.50). By contrast, each additional preventive medication received was negatively associated with patient satisfaction (AOR: 0.67, 95% CI: 0.48–0.95). Among facility-level variables, equipment availability was significant, with each equipment item associated with 10% increased odds of satisfaction (AOR: 1.10, 95% CI 1.01–1.21). No other facility variable, nor demographic variable, including parity, was statistically significant.

**Table 4:** Logistic regression analysis (final model) showing objective and subjective health system related factors evaluated for association with satisfaction with ANC, adjusted for sociodemographic factors, northern Nigeria, *N* = 1336

Independent variables	Adjusted Odds Ratio^a^	*p* value
Patient Socio-demography		
Age (years)	0.98 (0.94–1.03)	0.49
First Pregnancy		
Yes	1.11 (0.64–1.94)	0.71
No (ref)	1.00	
First ANC visit in facility for the pregnancy		
Yes	0.78 (0.45–1.36)	0.39
No (ref)	1.00	
Marital status		
Married/living-together	2.34 (.078–7.03)	0.13
Others (ref)	1.00	
Education		
Secondary or higher	0.96 (0.48–1.91)	0.91
Less than secondary (ref)	1.00	
Wealth quartile		
Poor	1.35 (0.68–2.66)	0.39
Lower-middle	1.67 (0.85–3.26)	0.13
Upper-middle (ref)	1.00	
Rich	1.29 (0.66–2.54)	0.45
Access to Care		
Out-of-pocket expenditure (Patient reported)		
Free care (ref)	1.00	0.01
Paid care	0.44 (0.23–0.82)	
Distance travelled in km (Patient reported)		
< 3	1.84 (0.42–8.16)	0.42
≥ 3 (ref)	1.00	
Patient Care Experience (Subjective perception)		
Non-discriminatory treatment regardless of socioeconomic status		
Yes	1.87 (1.09–3.22)	0.02
No (ref)	1.00	
Empathic providers		
Yes	1.82 (1.03–3.23)	0.01
No (ref)	1.00	
Assurance^b^	1.48 (1.26–1.75)	0.00
Responsiveness^c^	2.42 (2.05–2.87)	0.00
Treatment-facilitation^d^	2.03 (1.46–2.80)	0.00
Clinical examinations received^e^	1.28 (1.10–1.50)	0.00
Preventive medications received^f^	0.67 (0.48–0.95)	0.02
Facility Level Variable (Objectively measured by surveyors)		
Availability of general-care equipment^g^	1.10 (1.01–1.21)	0.00
Facility cleanliness and amenities	0.96 (0.87–1.07)	0.50
Availability of employed clinical staff on day of survey^h^	0.99 (0.98–1.01)	0.31

*ANC* Antenatal Care

^a^Adjusted for socio-demographic factors. None of the socio-demographic factors were significant. Significant at 0.05 level

^b^Assurance: Provider courtesy and accessibility, and trust in provider’s skill and treatment decisions

^c^ Responsiveness: Less wait time, adequate consultation time, respect for privacy, and clinic hours

^d^ Treatment-facilitation: Effective provider communication regarding maternal and fetal health condition and treatment, and ease of access to prescribed drugs

^e^Clinical examinations received: count of weight, height, blood pressure, uterine height, urine test, blood test, and abdominal examination received

^f^Prophylactic treatment: count of items received - iron/folic acid supplement, antimalarials, and tetanus toxoid

^g^Availability of general-care equipment: count of essential equipment available

^h^Clinical staff: Doctors, nurses, midwives, auxiliary nurse, pharmacists, laboratory technologists, technicians, community health officers, community health extension workers

## Discussion

The study purpose was to identify the modifiable factors associated with pregnant women’s satisfaction with ANC at government health facilities in Nigeria. First, removal of financial barriers (out-of-pocket payments) is important to pregnant women [14]. Secondly, we find an association between patient satisfaction and both the perceived quality of clinical aspects of care and interpersonal interactions of providers. Important clinical care quality factors were: patients trust in their providers’ medical decisions, and the number of clinical examinations patients received. Providers’ interpersonal interactions of significance were providers’ non-discriminatory behavior regardless of patient’s socioeconomic status, their concern for patients’ wellbeing (empathy), responsive provision of services (respect for patients’ time and privacy) and effective communication at consultation (a component of treatment facilitation). The facility’s status of essential equipment availability was also associated with patient satisfaction. Finally, medication logistics promoting ease of access to prescribed drugs at the facility was associated with patients’ satisfaction. The study identified a number of health system indicators needed improvement. There were widespread and major deficiencies in the availability of essential medical equipment and drugs, and the performance of essential clinical examination. A significant proportion of patients reported discriminatory behaviors based on patients’ socioeconomic status and un-empathic staff attitudes. Public health facilities require significant improvements to these aspects to render them truly functional, which may improve ANC utilization rate in Nigeria and may translate into a higher rate of institutional delivery.

We also identified a number of important factors relevant to ANC patient satisfaction that are not documented thus far. This study shows that out-of-pocket expenditure for ANC is of significant concern to patients in Nigeria, and this may be the case in similar low-income countries [14]. The ease of access to medicines at the facility may increase the likelihood of ANC users’ satisfaction with services. Poor medication access may reflect poor supervisory oversight resulting in unavailability of drugs and/or procedural bottlenecks to access available drugs [20].

Compared to earlier studies, our study tested a comprehensive range of patient-, provider- and facility-level factors associated with ANC patients’ satisfaction, and was therefore, well-positioned to robustly evaluate the role of these factors [15–19, 21]. Another strength of the study is the population based, large sample of both primary care and secondary level hospitals in rural and urban areas with an economically diverse patient clientele [15–19]. In addition, the proportion of patients excluded due to missing data (7%) was also quite low. Excluded patients were similar to the analytic sample on sociodemographic characteristics and satisfaction (table not shown). This study accounted for many structure and process variables impacting patients’ judgement of services (facility infrastructure, providers’ technical performance and interpersonal roles, and access to treatment) [21].

The observed negative association of out-of-pocket expenditure with satisfaction is consistent with other studies of family planning, and sexual health services [25, 26]. In low-income countries like Nigeria, among the poor population, the perceived benefits of ANC may be quite low compared to the opportunity cost in lost wages [14, 27]. Simulation studies have projected a significant increase in maternal healthcare utilization among the poor if user fees are abolished [28]. Our study provides empirical confirmation that, in order to achieve universal ANC coverage, it is important to offer it free of charge in poor communities. Our study evaluated the effect of any out-of-pocket expenses vs. none, supporting that all charges for ANC should be eliminated, along with effective facility oversight and supervisory measures to prevent informal fee demands by providers.

Notably, while the ease of medication access was positively associated with satisfaction (even when excluded from effective communication during consultation, and separately studied), the number of preventive medications received showed a negative association. More research is needed to explore the reasons for this contradictory finding, because of its potential consequence for medication compliance. Other studies corroborate the positive association of effective provider communication with satisfaction with ANC [16–18, 22, 23].Equipment availability at health facilities as verified by independent surveyors was independently associated with ANC patient satisfaction. The odds of patient satisfaction associated with this variable appears modest (a 10% increase in odds with each essential equipment item available), yet, when viewed against the prevailing equipment gap (17 out of 23 essential equipment unavailable, on average), the incremental odds translate into a significant role of equipment availability in patient satisfaction. Patients sense the lack of functional equipment and connect it with care quality [29]. Other studies are consistent with our findings: patients’ intent to return for care is associated with the adequacy of clinical examinations provided, and with providers’ ability to inspire confidence in their clinical care [7, 19, 22]. Courteous behavior is documented to increase ANC patient satisfaction [15–17]. Other studies also support our findings on the importance of responsive service (wait time and clinic hours), privacy during medical consultations (respecting patient dignity and confidentiality of medical information), adequate medical consultation time (unrushed service and alleviating patient concerns), empathic staff attitude, and non-discriminatory treatment regardless of socioeconomic status [15–17, 30].

### Limitations

One study limitation is the small number of patients interviewed per facility (2–3). No data were collected on the officially levied charges by the facility. Another limitation is the lack of data on provider-patient language concordance. Inability to communicate effectively with the patient due to language barrier may be miss-classified as an issue of interpersonal communication. Given the states for the NSHIP project were selected purposively from the Nigerian government’s perspective, the study findings may not be representative across Nigeria. We have no data on actual number of eligible ANC patients at the facilities visited and interview response rate.

## Conclusions

The study identified four modifiable factors associated with antenatal patients’ satisfaction with care; the availability of equipment and drugs, adequacy of clinical care, empathic and nondiscriminatory environment, and ease of access to treatment in healthcare facilities. These attributes fared poorly reflecting the poor state of the functioning health facilities in Nigeria. The policy implications of the study are to: equip health facilities with essential equipment and consumables, ensure provider adherence to national antenatal care guidelines, link provider compensation to the number of low-income patients served (to dis-incentivize discriminatory behavior toward disadvantaged patients), train and incentivize providers to engage in positive interactions with patients, establish a supervisory emphasis on a patient-centered care culture, optimize patient flow and medication logistics, expand free ANC clinics in poor communities, and lastly, monitor care quality through regular, anonymous patient satisfaction surveys. This strategic approach should potentially translate to increased ANC utilization, and in turn, high rates of institutional delivery and lower maternal mortality in Nigeria [9, 10].

## Additional file

Additional file 1: **Table S1.** Patient Satisfaction Survey**.** Pregnant Women’s Satisfaction with their Antenatal Care Visit - Survey Instrument**.** Details the survey items on the questionnaire used to interview the ANC outpatients and capture patient-level independent variables. (DOCX 15 kb)

## References

[1] National Population Commission, Nigeria, ICF International (2013). Nigeria demographic and health survey.

[2] World Bank. World databank; world development indicators. http://databank.worldbank.org/data/home.aspx. Accessed 20 April 2016.

[3] Dahiru T, Oche OM (2015). Determinants of antenatal care, institutional delivery and postnatal care services utilization in Nigeria. Pan Afr Med J.

[4] Pervin J, Moran A, Rahman M, Razzaque A, Sibley L, Streatfield PK (2012). Association of antenatal care with facility delivery and perinatal survival - a population-based study in Bangladesh. BMC Pregnancy Childbirth.

[5] Ntambue AM, Malonga FK, Dramaix-Wilmet M, Ngatu RN, Donnen P (2016). Better than nothing? Maternal, newborn, and child health services and perinatal mortality, Lubumbashi, democratic republic of the Congo: a cohort study. BMC Pregnancy Childbirth..

[6] Schempf AH, Minkovitz CS, Strobino DM, Guyer B (2007). Parental satisfaction with early pediatric care and immunization of young children: the mediating role of age-appropriate well-child care utilization. Arch Pediatr Adolesc Med.

[7] Rani M, Bonu S, Harvey S (2008). Differentials in the quality of antenatal care in India. Int J Qual Health Care.

[8] World Health Organization, United Nations Children’s Fund, United Nations Population Fund, World Bank Group, United Nations Population Division. Trends in maternal mortality: 1990 to 2015. http://www.who.int/reproductivehealth/publications/monitoring/maternal-mortality-2015/en/. Accessed 20 Apr 2016.

[9] Ministry of Health and Family Welfare, India. Maternal health Programme. https://mohfw.gov.in/sites/default/files/Chapter415.pdf. Accessed 3 Mar 2016.

[10] Powell-Jackson T, Mazumdar S, Mills A (2015). Financial incentives in health: new evidence from India's Janani Suraksha Yojana. J Health Econ.

[11] Lin H, Xirasagar S, Laditka J (2004). Patient perceptions of service quality in group versus solo practice clinics. Int J Qual Health Care.

[12] Rao KD, Peters DH, Bandeen-Roche K (2006). Towards patient-centered health services in India—a scale to measure patient perceptions of quality. Int J Qual Health Care.

[13] Nabbuye-Sekandi J, Makumbi FE, Kasangaki A, Kizza IB, Tugumisirize J, Nshimye E (2011). Patient satisfaction with services in outpatient clinics at Mulago hospital, Uganda. Int J Qual Health Care.

[14] Finlayson K, Downe S (2013). Why do women not use antenatal services in low- and middle-income countries? A meta-synthesis of qualitative studies. PLoS Med.

[15] Vo BN, Cohen CR, Smith RM, Bukusi EA, Onono MA, Schwartz K (2012). Patient satisfaction with integrated HIV and antenatal care services in rural Kenya. AIDS Care.

[16] Wong ST, Korenbrot CC, Stewart AL (2004). Consumer assessment of the quality of interpersonal processes of prenatal care among ethnically diverse low-income women: development of a new measure. Womens Health Issues.

[17] Korenbrot CC, Wong ST, Stewart AL (2005). Health promotion and psychosocial services and women's assessments of interpersonal prenatal care in Medicaid managed care. Matern Child Health J.

[18] Ohnishi M, Nakamura K, Takano T (2007). Training of healthcare personnel to improve performance of community-based antenatal care program. Adv Health Sci Educ Theory Pract.

[19] Smith LF (1999). The WOMB (Women's views of birth) antenatal satisfaction questionnaire: development, dimensions, internal reliability, and validity. Br J Gen Pract.

[20] Cham M, Sundby J, Vangen S (2009). Availability and quality of emergency obstetric care in Gambia's main referral hospital: women-users’ testimonies. Reprod Health.

[21] Donabedian A (1988). The quality of care; how can it be assessed. JAMA.

[22] Abioye Kuteyi EA, Bello IS, Olaleye TM, Ayeni IO, Amedi MI (2010). Determinants of patient satisfaction with physician interaction: a cross-sectional survey at the Obafemi Awolowo university health Centre, Ile-Ife, Nigeria. S Afr Fam Pract.

[23] Wachira J, Middlestadt S, Reece M, Peng CJ, Braitstein P (2014). Physician communication behaviors from the perspective of adult HIV patients in Kenya. Int J Qual Health Care.

[24] Marchenko Y. Multiple-imputation analysis using Stata’s mi command. Boston: Paper presented at the Stata Conference; 2010. July 16. http://citeseerx.ist.psu.edu/viewdoc/download?doi=10.1.1.473.1054&rep=rep1&type=pdf. Accessed 20 Apr 2016.

[25] Agha S, Do M (2009). The quality of family planning services and client satisfaction in the public and private sectors in Kenya. Int J Qual Health Care.

[26] Meuwissen LE, Gorter AC, Knottnerus JA (2006). Perceived quality of reproductive care for girls in a competitive voucher programme. A quasi-experimental intervention study, Managua, Nicaragua. Int J Qual Health Care.

[27] National Bureau of Statistics. Nigeria poverty profile 2010. http://www.nigerianstat.gov.ng/pdfuploads/Nigeria%20Poverty%20Profile%202010.pdf. Accessed 13 June 2016.

[28] Hotchkiss DR, Krasovec K, El-Idrissi MDZ, Eckert E, Karim AM (2005). The role of user charges and structural attributes of quality on the use of maternal health services in Morocco. Int J Health Plann Manag.

[29] Nigenda G, Langer A, Kuchaisit C, Romero M, Rojas G, Al-Osimy M (2003). Womens’ opinions on antenatal care in developing countries: results of a study in Cuba, Thailand. Saudi Arabia and Argentina. BMC Public Health.

[30] Hearld LR, Alexander JA (2012). Patient-centered care and emergency department utilization: a path analysis of the mediating effects of care coordination and delays in care. Med Care Res Rev.

