# Supplementary material for: Antenatal care satisfaction in a developing country: a cross-sectional study from Nigeria
Source: BMC Public Health. 2018 Mar 20;18:368. doi: 10.1186/s12889-018-5285-0 (PMC5859482; doi:10.1186/s12889-018-5285-0)
Supplement: Supplementary file 1 — Table S1. Patient Satisfaction Survey. Pregnant Women’s Satisfaction with their Antenatal Care Visit - Survey Instrument. Details the survey items on the questionnaire used to interview the ANC outpatients and capture patient-level independent variables. (DOCX 15 kb) [file 12889_2018_5285_MOESM1_ESM.docx]

| **Table S1: Pregnant Women’s Satisfaction with their Antenatal Care Visit - Survey Instrument** | | | |
| --- | --- | --- | --- |
| (1) | IDENTIFICATION AND DEMOGRAPHIC CHARACTERISTICS | | Record Response |
| 1.01 | Patient Name | |  |
| 1.02 | Patient Age (years) | |  |
| 1.03 | What is the highest education you ever attended | Pre-primary 0 |  |
|  |  | Primary 1 |  |
|  |  | Secondary 2 |  |
|  |  | Higher 3 |  |
| 1.04 | Highest class completed within the level |  |  |
| 1.05 | What is your marital status | Single 1 |  |
|  |  | Married/living together 2 |  |
|  |  | Widowed 3 |  |
|  |  | Divorced/separated 4 |  |
| 1.06 | Is this your first pregnancy | Yes 1 |  |
|  |  | No 2 |  |
| 1.07 | Is this your first antenatal visit at this facility for this pregnancy | Yes 1 |  |
|  |  | No 2 |  |
| (2) | TREATMENT | |  |
| 2.01 | During this visit, were you weighed? | Yes 1 |  |
|  |  | No 2 |  |
| 2.02 | During this visit, was your height measured? | Yes 1 |  |
|  |  | No 2 |  |
| 2.03 | During this visit, did someone measure your blood pressure? | Yes 1 |  |
|  |  | No 2 |  |
| 2.04 | During this visit, did you give a urine sample? | Yes 1 |  |
|  |  | No 2 |  |
| 2.05 | During this visit, did you give a blood sample? | Yes 1 |  |
|  |  | No 2 |  |
| 2.06 | During this visit, did the provider palpate your tummy? | Yes 1 |  |
|  |  | No 2 |  |
| 2.07 | During this visit, was your uterine height  measured? | Yes 1 |  |
|  |  | No 2 |  |
| 2.08 | During this visit, did a health worker give you iron pills, folic acid or iron with folic acid, or give you a prescription for them? | Yes 1 |  |
|  |  | No 2 |  |
| 2.09 | ASK TO SEE THE CLIENT’S IRON/FOLIC  ACID/IRON WITH FOLIC ACID PILLS OR  PRESCRIPTION FOR IT. | Saw Pills 1 |  |
|  |  | Saw Prescription 2 |  |
|  |  | No Pills or Prescription 3 |  |
| 2.10 | During this visit, has a health worker given or prescribed any antimalarial pills for you? | Yes 1 |  |
|  |  | No 2 |  |
| 2.11 | ASK TO SEE THE CLIENT’S ANTIMALARIAL  PILLS OR PRESCRIPTION FOR IT. | Saw Pills 1 |  |
|  |  | Saw Prescription 2 |  |
|  |  | No Pills or Prescription 3 |  |
| 2.12 | During this visit or previous visits, has a health worker asked you whether you had ever received a tetanus toxoid injection? | Yes 1 |  |
|  |  | No 2 |  |
| 2.13 | Have you ever received a tetanus toxoid injection, including one you may have received today? | Yes 1 |  |
|  |  | No 2 |  |
| (3) | COUNSELLING |  |  |
| 3.01 | During this visit, did a health worker give you advice on your diet? | Yes 1 |  |
|  |  | No 2 |  |
| 3.02 | During this visit or previous visits, has a health worker talked with you about any signs of complications (danger signs) that should warn you of problems with the pregnancy? | Yes, during this visit 1 |  |
|  |  | Yes, previous visit 2 |  |
|  |  | No 3 |  |
| 3.03 | During this visit, did a health worker talk with you about using family planning after the birth of your baby? | Yes 1 |  |
|  |  | No 2 |  |
| 3.04 | During this visit or previous visits, has a provider given you advice on the importance of exclusively breastfeeding | Yes, during this visit 1 |  |
|  |  | Yes, previous visit 2 |  |
|  |  | No 3 |  |
| 3.05 | During this visit or previous visits, did the provider talk to you about where you plan to deliver your baby? | Yes, during this visit 1 |  |
|  |  | Yes, previous visit 2 |  |
|  |  | No 3 |  |
| 3.06 | During this or previous visits, did a provider talk with you about HIV counseling and testing? | Yes, during this visit 1 |  |
|  |  | Yes, previous visit 2 |  |
|  |  | No 3 |  |
| (4 ) | TRAVEL AND EXPENDITURE | |  |
| 4.01 | How far is your household from this health facility? | Kilometers |  |
| 4.02 | Did you have to pay a registration, consultation or doctor's fee? | Yes 1 |  |
|  |  | No 2 |  |
| 4.03 | How much did you pay for this in Naira? | NAIRA |  |
| 4.04 | Was a laboratory test done? | Yes 1 |  |
|  |  | No 2 |  |
| 4.05 | How much was paid in Naira for lab tests? | NAIRA |  |
| 4.06 | Was an ultrasound done? | Yes 1 |  |
|  |  | No 2 |  |
| 4.07 | How much was paid in Naira for ultrasound? | NAIRA |  |
| 4.08 | Were medicines dispensed to you today? | Yes 1 |  |
|  |  | No 2 |  |
| 4.09 | How much was paid in Naira for medicines? | NAIRA |  |
| 4.10 | Did you give any informal gift or gratification to the health worker(s)? | Yes 1 |  |
|  |  | No 2 |  |
| 4.11 | How much money was given or what is the money value of the gift? | NAIRA |  |
| (5) | PATIENT SATISFACTION | Agree 3 |  |
|  |  | Neutral 2 |  |
|  |  | Disagree 1 |  |
| 5.01 | The health staff are courteous and respectful | |  |
| 5.02 | The health workers did a good job of explaining your health condition. | |  |
| 5.03 | It is easy to get medicine that health workers prescribe. | |  |
| 5.04 | The amount of time you spent waiting to be seen by a health provider was reasonable. | |  |
| 5.05 | You had enough privacy during your visit. | |  |
| 5.06 | The health worker spent a sufficient amount of time with you | |  |
| 5.07 | The hours the facility is open are adequate to meet your needs | |  |
| 5.08 | The health workers in this facility are extremely thorough and careful. | |  |
| 5.09 | You trust in the skills and abilities of the health workers of this facility. | |  |
| 5.10 | You completely trust the health worker’s decisions about medical treatments in this facility. | |  |
| 5.11 | The health workers in this facility are very friendly and approachable. | |  |
| 5.12 | The health workers in this facility are easy to make contact with. | |  |
| 5.13 | The health workers in this facility care about your health just as much or more than you do. | |  |
| 5.14 | The health workers in this facility act differently toward rich people than toward poor people. | |  |
